# Asymmetric Periodic Boundary Conditions for All-Atom Molecular Dynamics and Coarse-Grained Simulations of Nucleic Acids

**DOI:** 10.1021/acs.jpcb.3c03887

**Published:** 2023-09-15

**Authors:** Radek Erban, Yuichi Togashi

**Affiliations:** † Mathematical Institute, University of Oxford, Radcliffe Observatory Quarter, Woodstock Road, Oxford OX2 6GG, U.K.; ‡ Department of Bioinformatics, College of Life Sciences, Ritsumeikan University, 1-1-1 Noji-higashi, Kusatsu, Shiga 525-8577, Japan; § RIKEN Center for Biosystems Dynamics Research, 2-2-3 Minatojima-Minamimachi, Chuo-ku, Kobe, Hyogo 650-0047, Japan

## Abstract

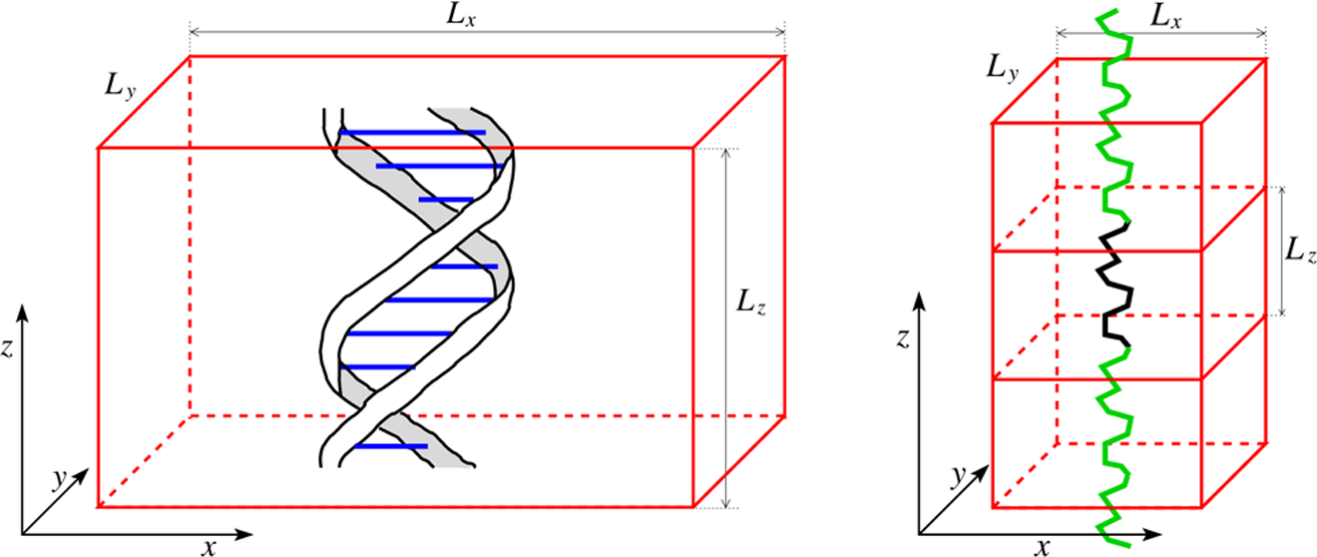

Periodic boundary conditions are commonly applied in molecular dynamics simulations in the microcanonical (NVE), canonical (NVT), and isothermal–isobaric (NpT) ensembles. In their simplest application, a biological system of interest is placed in the middle of a solvation box, which is chosen ‘sufficiently large’ to minimize any numerical artifacts associated with the periodic boundary conditions. This practical approach brings limitations to the size of biological systems that can be simulated. Here, we study simulations of effectively infinitely long nucleic acids, which are solvated in the directions perpendicular to the polymer chain, while periodic boundary conditions are also applied along the polymer chain. We study the effects of these asymmetric periodic boundary conditions (APBC) on the simulated results, including the mechanical properties of biopolymers and the properties of the surrounding solvent. To get some further insights into the advantages of using the APBC, a coarse-grained worm-like chain model is first studied, illustrating how the persistence length can be extracted from the local properties of the polymer chain, which are less affected by the APBC than some global averages. This is followed by all-atom molecular dynamics simulations of DNA in ionic solutions, where we use the APBC to investigate sequence-dependent properties of DNA molecules and properties of the surrounding solvent.

## 1 Introduction

The structure and function of DNA depend on its nucleotide sequence and on the properties of the surrounding solvent.^1^ Since DNA is negatively charged, concentrations of ions are perturbed from their bulk values in the region close to DNA. The resulting ‘ion atmosphere’ has been studied using ion counting experiments.^2^ From the theoretical point of view, all-atom molecular dynamics (MD) simulations can be applied to provide detailed insights into DNA, ions, and water interactions.^3^ For example, the effect of mobile counterions, Na^+^ and K^+^, on a DNA oligomer was studied by Várnai and Zakrzewska,^4^ who used periodic boundary conditions for MD simulations of the solvated DNA oligomer at constant temperature and pressure and studied the counterion distribution around the DNA structure.

However, the applicability of all-atom MD studies is limited to relatively small systems. To simulate larger systems, several coarse-grained approaches have been developed in the literature. In adaptive resolution studies,^5,6^ DNA and its immediate neighborhood are simulated using the full atomistic resolution, while a coarse-grained description is used to describe the solvent molecules that are far away from DNA. Solvent can also be treated implicitly in faraway regions.^6^ To model even larger systems, the DNA molecule itself can be described by coarse-grained models.^7−10^ Examples vary from models using several coarse-grained sites per nucleotide^11^ to Brownian dynamics simulations.^12,13^ Using a systematic ‘bottom-up approach’, the interaction potential between coarse-grained sites can be derived from the underlying atomistic force field, with results dependent on the microscopic force field used.^14^

**Figure 1:**
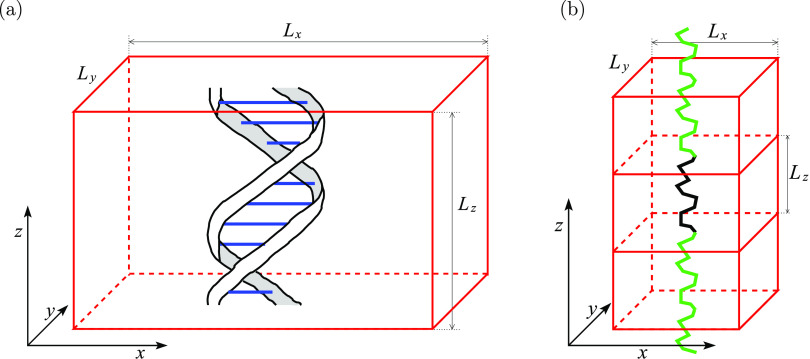
(a) Schematic of the simulation domain given by eq 1 for the case with 10 base pairs of DNA, i.e., for *n* = 1. (b) Discrete worm-like chain segment in an APBC simulation is denoted by the black line. Its periodic image copies are visualized as the green lines.

The properties of ions in bulk water can be studied using all-atom MD simulations,^15,16^ which provide ‘bottom-up’ estimates of the values of diffusion constants of ions. Some biological processes include transport of ions across relatively large distances which are out of reach to all-atom MD simulations. Brownian dynamics descriptions of ions are instead used for modeling such systems.^17,18^ While the transport of ions in bulk water can be described on a sufficiently long time scale as a standard Brownian motion, more detailed coarse-grained stochastic models of ions in bulk water have to be used at time scales studied by MD simulations.^16^ Coarse-grained stochastic models of ions can be written as systems of stochastic differential equations or by the generalized Langevin equation.^16,19^ To parametrize such models, detailed all-atom MD simulations of ‘long’ chains of nucleic acids can be used, but this can be computationally intensive. In this paper, we investigate simulations of effectively infinitely long DNA by applying asymmetric periodic boundary conditions (APBC) in the cuboid computational domain
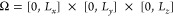
1The main idea behind the APBC is that DNA is periodic with the period of 10 base pairs, i.e., the APBC will allow us to use 10*n* base pairs of DNA in domain Ω, where  is an integer denoting the number of helical pitches. A schematic of our simulation domain is presented in Figure 1a for the case of the simulation with 10 base pairs, i.e., for *n* = 1. The DNA molecule is positioned parallel to the *z*-axis, and we use periodic boundary conditions in the *z*-direction. Such a periodic boundary condition in the *z*-direction is less common in all-atom MD simulation studies, where the biomolecule of interest is often placed in the middle of the computational domain and it is solvated on all its sides by a layer of water molecules separating the biomolecule from the domain boundary.^4,20^

Considering the projection into the *xy*-plane, the DNA molecule is positioned in the middle of the simulated domain. In particular, the DNA molecule is separated by the layer of water molecules from the boundaries of the simulated domain in both the *x*-direction and *y*-direction. While we use periodic boundary conditions in all three directions, there is an asymmetry (highlighted in our terminology APBC): a modeler has a relative freedom to choose the values of *L*_*x*_ and *L*_*y*_ in the computational domain defined by eq 1, while the value of *L*_*z*_ is dictated by the properties of the simulated biomolecule. The imposed DNA periodicity fixes the helical twist of the DNA molecule with the simulation box size *L*_*z*_ chosen such that it exactly corresponds to *n* helical pitches. However, considering simulations at isothermal–isobaric (NpT) ensemble, standard isotropic barostats introduce fluctuations in the domain size leading to changes in *L*_*z*_ as well. To fix *L*_*z*_, an asymmetric barostat is used in Section 3.

The APBC has been used in previous studies^5,21,22^ to simulate effectively infinitely long DNA molecules. Except for the asymmetry between the *z*-direction and *x*-direction (resp. *y*-direction), the APBC can lead to a relatively standard all-atom MD setup, with the domain periodic in all three directions, which was previously used to explore the ion atmosphere around the DNA.^21,22^ However, it is more challenging to use the APBC to study the mechanical properties of biopolymers, as we will first illustrate in Section 2 by considering a discrete worm-like chain model. This is followed by all-atom MD simulations of DNA in Section 3, where we present the use of APBC to investigate the mechanical properties of the DNA and the properties of the surrounding solvent.

## 2 Worm-like Chain Model

Let us consider the discrete worm-like chain (WLC) model where DNA consists of *N* segments **l**_*i*_ , *i* = 1, 2, ..., *N*, each having the same length, δ. Denoting the angle between the adjacent *i*th and (*i* + 1)th segments by θ_*i*_, for *i* = 1, 2, ..., (*N* – 1), the chain bending energy of the discrete WLC model is
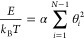
2where α is a dimensionless constant. The sum on the right-hand side of eq 2 corresponds to an integral term in the continuous WLC model,^23,24^ and eq 2 can be also derived by discretizing this integral term.^1^ Then, one can also establish that our dimensionless stiffness parameter α is proportional to the persistence length of the continuous WLC model.^24^ This is also true for the discrete WLC model in the limit α → ∞, where we get the persistence length equal to 2δα to the leading order (see eq 8). To show this, we define the persistence length of the first *j*th segments of the discrete WLC model, for *j* ≤ *N*, by
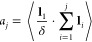
3That is, *a*_*j*_ is the average value of the projection of the vector connecting the end points of the first and the *j*th segment on the direction of the first segment. Then, the persistence length of the discrete WLC model can be defined as the limit
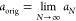
4which effectively is the average value of the projection of the end-to-end vector of a long chain in the direction of the first segment. In this paper, we will only study the discrete version of the WLC model because it will provide more insight into the use of the APBC than the continuous model. In particular, in the following, the WLC model means the discrete WLC model with the energy defined by eq 2.

### 2.1 Dependence of Persistence Length on Stiffness Parameter α

**Figure 2:**
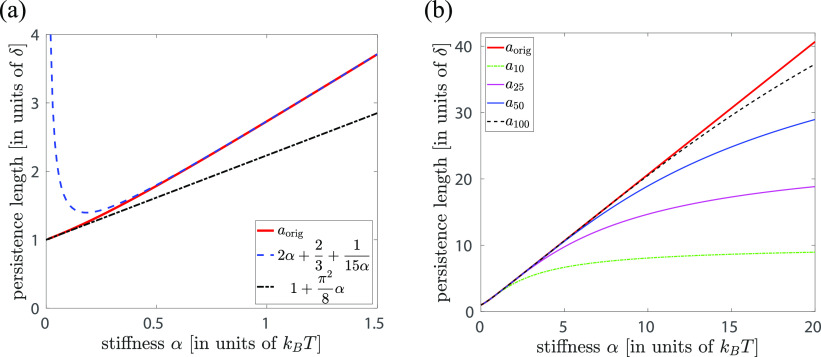
(a) Plot of persistence length *a*_orig_, given by eq 4, as a function of the stiffness parameter α, together with asymptotic results given by eqs 8 and 9. The dimensionless parameter α can be viewed to express energy in units *k*_*B*_*T*, while all persistence lengths are plotted in units of the segment length, δ. (b) Plot of persistence length *a*_orig_, given by eq 4, and persistence lengths *a*_*j*_, given by eq 5, for *j* = 10, 25, 50, 100, as a function of parameter α.

The average in eq 3 can be evaluated as
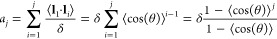
5where the average ⟨cos(θ)⟩ = ⟨cos(θ_*i*_)⟩ is independent of the index *i* because the chain bending energy in eq 2 is symmetric with respect to any permutation of indices. Using spherical polar coordinates,^25^ we have
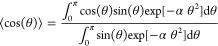
6To get formula 6, we note that the distribution of angles between adjacent segments is proportional to sin(θ)exp[−αθ^2^]. Using eqs 4, 5, and 6, we deduce (see Appendix A.1)
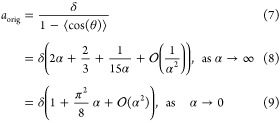
In Figure 2a, we present how the persistence length *a*_orig_ depends on the stiffness parameter α in interval [0, 3/2], illustrating the accuracy of both expansions in eqs 8 and 9. While eq 8 is derived in the limit α → ∞, it approximates the exact result well for persistence lengths satisfying  or equivalently for α > 0.62. In Figure 2b, we plot the dependence of the persistence length *a*_orig_ on the stiffness parameter α in a larger interval [0, 20] together with the values of *a*_*j*_ given by eq 5. Using the exact result for *a*_orig_ given in eq 7, we can rewrite eq 5 as follows
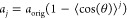
10Considering the limit α → ∞ in eq 6, we have
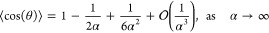
where the first three terms of the expansion on the right-hand side provide an approximation of ⟨cos(θ)⟩ with about 5% relative error for α > 1, and the relative error decreases as we increase the value of α, for example, the relative error is smaller than 1% for α > 2. Substituting this expansion for ⟨cos(θ)⟩ into eq 10, we obtain that for sufficiently large values of α, say for α > 1, we can calculate the persistence length *a*_orig_ from *a*_*j*_ by using the following formula
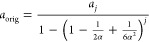
11

### 2.2 Dependence of Persistence Length on APBC

**Figure 3:**
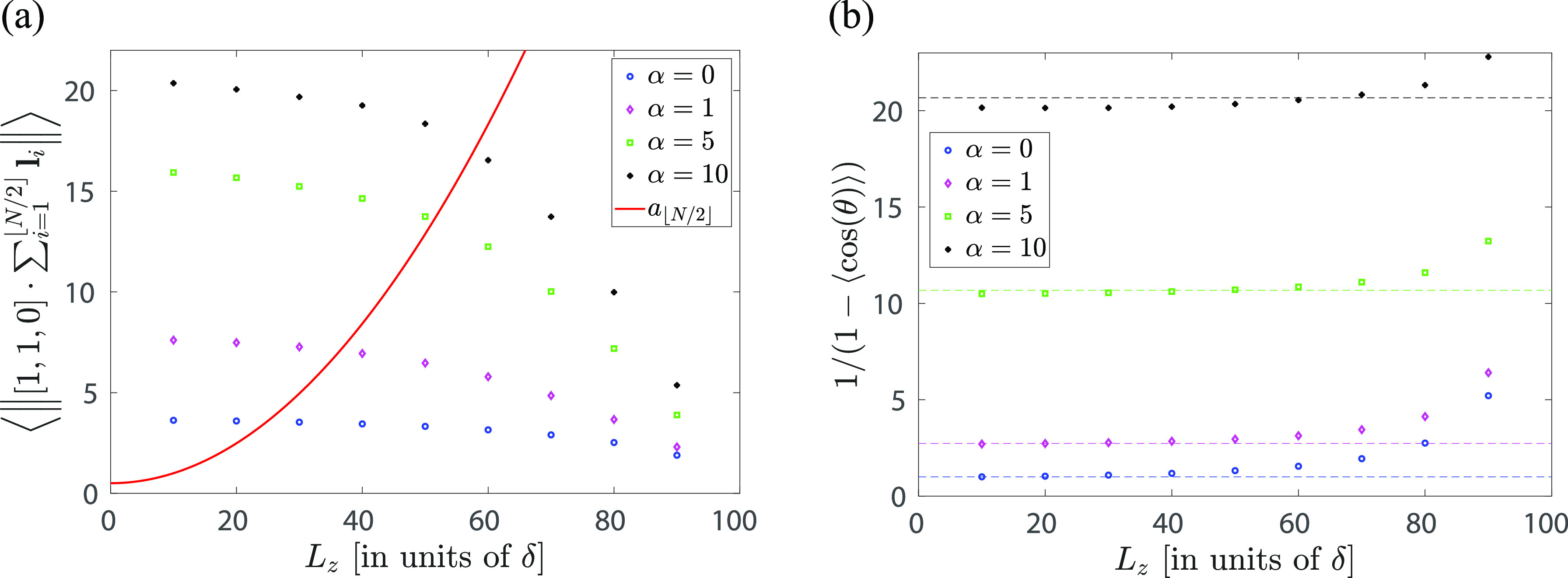
(a) Average distance of the polymer middle point from the axis of the polymer, defined by eq 14, estimated from Monte Carlo simulations of the WLC model with *N* = 100 segments for *L*_*z*_ ∈ {10, 20, ..., 90} and α ∈ {0, 1, 5, 10}. The red line shows  as a function of the domain length *L*_*z*_ (theoretical result given by eq 15 confirmed by simulations for all considered values of α). Further details of Monte Carlo simulations are in Appendix A.3. (b) The estimate of *a*_orig_ given by eq 16, where ⟨cos(θ)⟩ is estimated from Monte Carlo simulations of the WLC model with *N* = 100 segments for *L*_*z*_ ∈ {10, 20, ..., 90} and α ∈ {0, 1, 5, 10}. The theoretical result (without APBC, independent of *L*_*z*_), given by eq 6, is plotted by dashed lines for each value of α.

Considering that the polymer chain is simulated in the domain given by eq 1 with APBC, we have an extra constraint
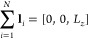
12where *N* denotes the number of simulated segments along the *z*-direction. As illustrated in Figure 1b, such a model can be viewed as a model of an (infinitely) long polymer chain by using the periodicity
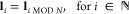
13However, substituting eqs 12 and 13 into the definition of persistence length in eq 4, we would obtain that *a*_orig_ = ∞ because the periodic boundary means that the infinitely long filament is effectively straight. Since eq 12 postulates that the vector connecting ends of *N* segments is fixed, we obtain the most variability in this model by looking at the behavior of the  consecutive segments. Due to the symmetry of the problem and eq 12, the average of the vector  is equal to [0, 0, *L*_*z*_/2] for any value of α, but the deviations from this average will be larger when we increase the stiffness parameter α. To illustrate this, we define the average distance of the polymer middle point from the axis of the polymer by
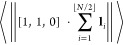
14that is, we calculate the (Euclidean) norm of the projection of the vector  on the *x*–*y* plane. The average in eq 14 is plotted in Figure 3a for different values of parameter α and domain length *L*_*z*_. We observe that, for a fixed value of *L*_*z*_, the average in eq 14 increases with the value of the stiffness parameter α. Moreover, Figure 3a also shows that the value of the average in eq 14 approaches zero as *L*_*z*_ approaches its maximum possible value, *N*δ. Indeed, if , the polymer is straight and the value of the average in eq 14 is exactly equal to zero. On the other hand, if *L*_*z*_ is smaller, then we obtain a larger value of the average in eq 14, especially for polymers with larger persistence lengths (i.e., for large values of α).

On the face of it, one possible way to estimate *a*_orig_ could be to estimate  from our Monte Carlo simulations^26^ and then use formula 11 for . However, formula 11 has been derived for the case of the WLC model in the three-dimensional physical space . Considering the APBC, we obtain that  is independent of α (see Appendix A.2). We have
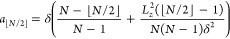
15which simplifies to  for large values of *N*. This result is also visualized in Figure 3a. In particular, a better strategy to obtain the real persistence length *a*_orig_ from the APBC simulations is to estimate
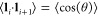
and then use the exact result for *a*_orig_ given in eq 7, namely
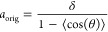
16The results are presented in Figure 3b. We observe that the results given by eq 16, where ⟨cos(θ)⟩ is estimated from Monte Carlo simulations (with APBC), agree well (especially for smaller values of *L*_*z*_) with the theoretical result, which has been obtained without the use of APBC in eq 6. As we increase *L*_*z*_, the error of this estimation increases. In fact, if , then the WLC polymer becomes a straight line, giving ⟨cos(θ)⟩ = 1 and formula 16 diverges, as we can also observe in Figure 3b for larger values of *L*_*z*_.

## 3 APBC in All-Atom MD Simulations

In this section, we investigate the use of APBC in all-atom MD models of DNA. Our simulations are performed with 10–100 base pairs (bp) of double-stranded DNA (dsDNA). Since we use APBC, all simulations are effectively simulating (infinitely) long DNA chains. In particular, MD results with the longest simulated chain (100 bp) can be used as the ‘ground truth’ for the presented APBC simulations with shorter 10–50 bp long DNA chains. We note that the MD simulations of relatively short 50 bp DNA segments without APBC have been previously used in the literature to estimate the DNA persistence length by using a middle section of the simulated DNA segment.^20,27^

We consider 6 types of (infinitely) long DNA sequences, with repeated nucleotides, namely, poly(A), poly(C), poly(AT), poly(CG), poly(AC), and poly(AG), where poly(X) means that the corresponding nucleotide sequence is periodically repeated. We note that these 6 cases correspond to all possible cases of pairs of nucleotides which are repeated infinitely many times. For example, repetitions of dinucleotides AC, CA, TG, and GT all correspond to the poly(AC) case because AC and CA are equivalent due to the periodic boundary conditions along the chain length, and TG is on the complementary strand, with GT being equivalent to TG because of the periodic boundary conditions.

**Figure 4:**
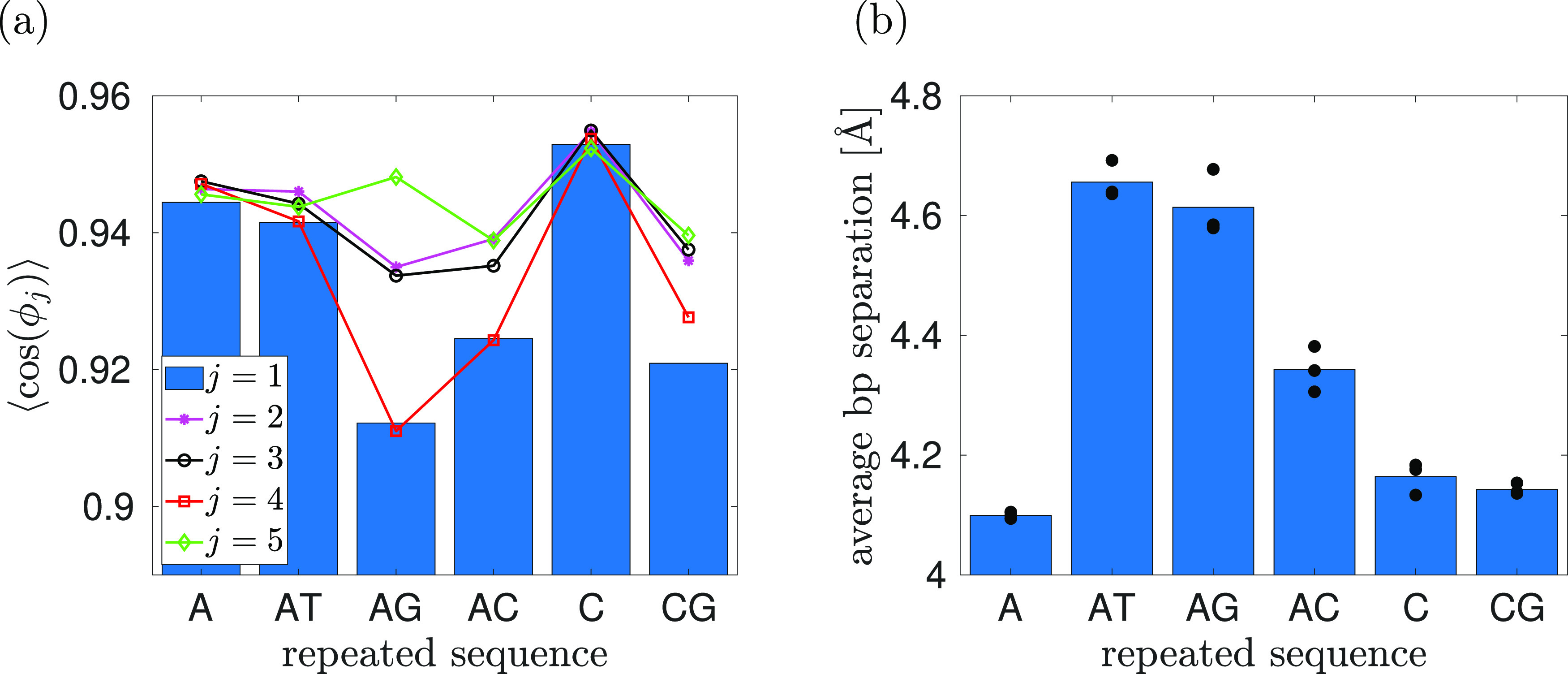
Results of all-atom MD simulations of DNA chains with APBC and *N* = 10. (a) The average given by eq 18 for each of the 6 considered sequences of repeated nucleotides is calculated using three independent MD simulations. (b) The average separation between the base pairs calculated using three independent MD simulations (blue bars). The results for each individual realization are plotted as a black dot.

**Figure 5:**
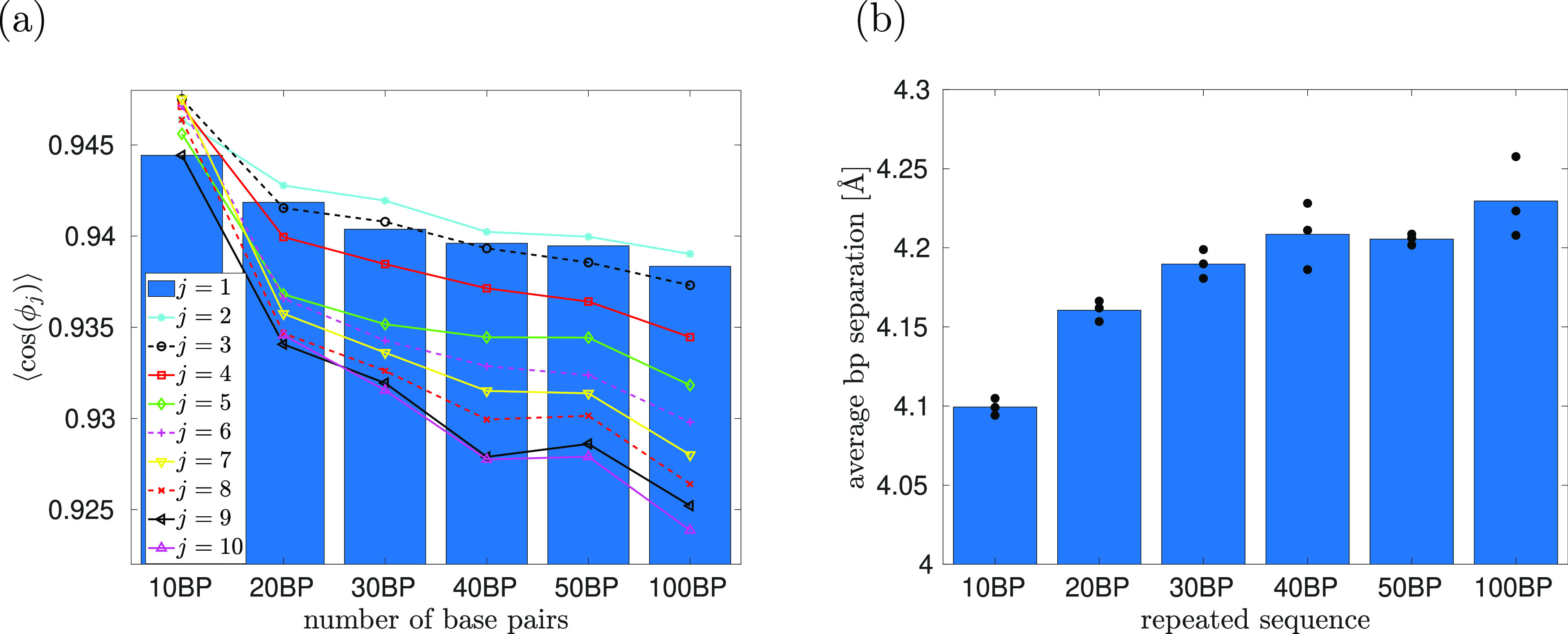
Results of all-atom MD simulations with APBC using the poly(A) DNA chain with *N* in the range 10–100 bp. (a) The average given by eq 18 for each of the 6 values of *N* considered is calculated using three independent MD simulations. The results are presented for *j* = 1, 2, ..., 10 and *n* = 1, 2, 3, 4, 5, 10. (b) The average separation between the base pairs calculated using three independent MD simulations (blue bars). The results for each individual realization are plotted as a black dot.

**Figure 6:**
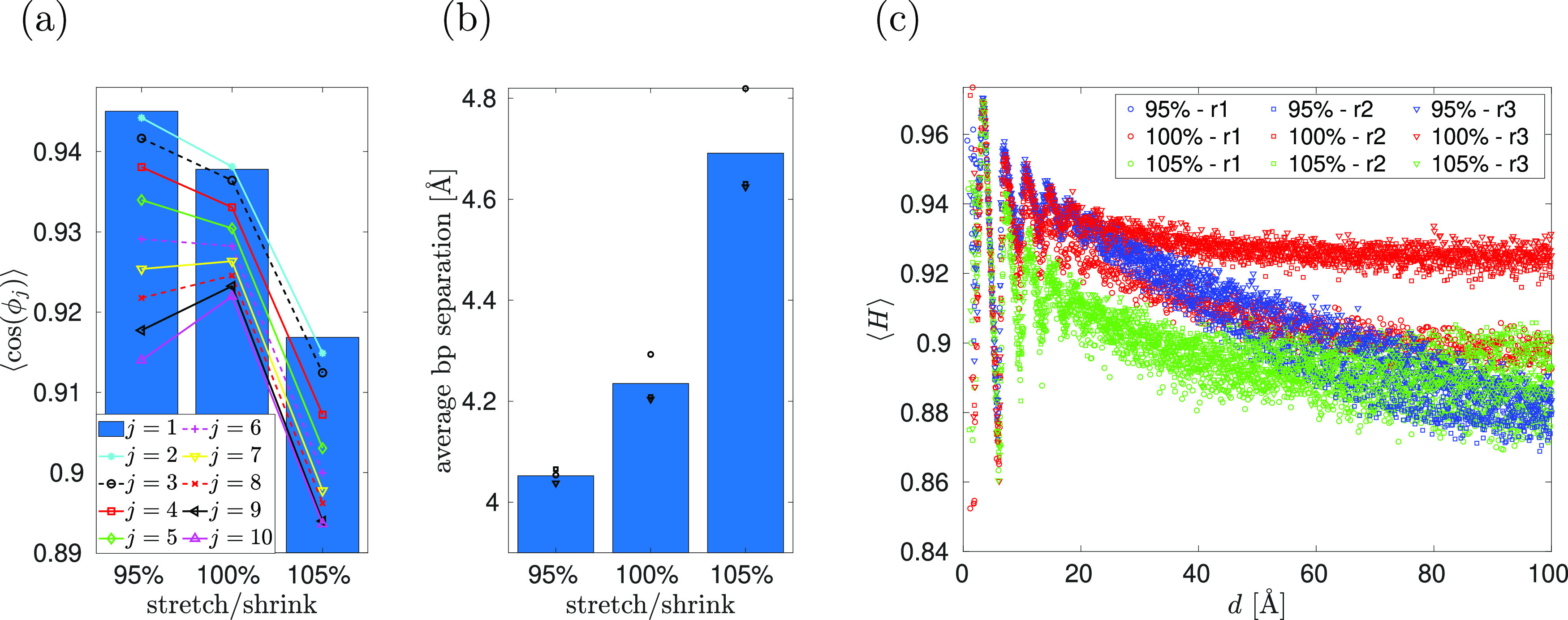
Results of MD simulations of 100 bp poly(A) dsDNA with APBC that use the values of *L*_*z*_ given by eq 19. We average over three independent MD time series for each of the presented case. (a) The average in eq 18 for *j* = 1, 2, ..., 10. (b) The average separation of base pairs (blue bars). Dots include the results for individual MD realizations (i.e., we have averaged over the dots to calculate blue bars). (c) The average ⟨*H*⟩ as a function of distance *d*. We present results for the 95% (blue), 100% (red), and 105% (green) cases using different colors. Different symbols (circle, square, triangle of the same color) denote data points calculated by different MD realizations.

**Figure 7:**
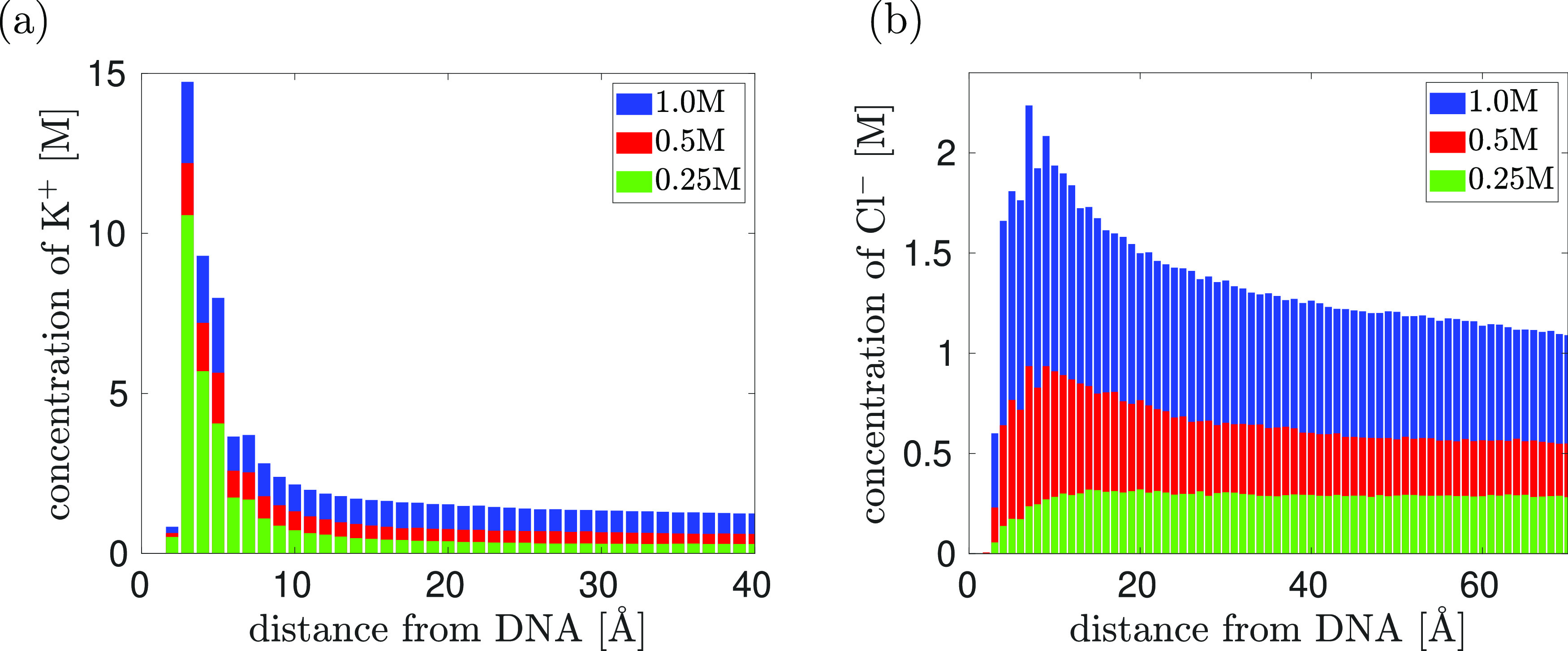
Results of all-atom MD simulations with APBC using the poly(A) DNA chain with *N* = 10 bp and three different concentrations of KCl in the bulk. (a) The concentration of K^+^ ions given by eq 21 as a function of the distance from DNA. (b) The concentration of Cl^–^ ions given by eq 21 as a function of the distance from DNA.

Each infinitely long sequence is modeled in our computational domain given by eq 1 with APBC using *N* = 10*n* base pairs of DNA, where *n* ranges from 1 to 10. The APBC is implemented along the *z*-direction as it is detailed in Appendix B.1. First, an (*N* + 1) bp long dsDNA configuration is constructed in such a way that the (*N* + 1)th base pair is equivalent to the first base pair translated to the *z*-direction. Then, a nucleotide at the 3’-end of each strand is removed and the bond to the 3’-end (removed) nucleotide is substituted with that to the first base at the 5’-end. The corresponding angles and dihedrals are added to MD structural files as detailed in Table 1 in Appendix B.1. In all MD simulations, we consider domain in eq 1 with *L*_*x*_ = *L*_*y*_ = 200 Å and we vary *L*_*z*_. In Figures 4, 5, and 7, we choose *L*_*z*_ as a multiple of *n* (resp. *N*) with
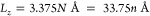
17while we study the effect of stretching and shrinking of DNA in Figure 6 by using *L*_*z*_ obtained as the 95, 100, and 105% of the value given by eq 17. All MD simulations are done in KCl solutions, with K^+^ ions neutralizing the negatively charged DNA segments. We use the concentration 150 mM KCl in Figures 4–6, while we vary the concentration of KCl in Figure 7.

When using APBC with polymer models, there are (locally) two important directions: parallel to the polymer chain and perpendicular to the polymer chain. We consider both of them, in Sections 3.1 and 3.2, respectively. In Section 3.1, we study the effects of APBC on the properties of the DNA chain, where we can make direct analogues to the results obtained for the persistence length of the WLC model in Section 2. This is followed by studying the characteristics of the surrounding solvent in Section 3.2, where we investigate the ion atmosphere around DNA for different concentrations of KCl.

### 3.1 Dependence of the Polymer Chain Properties on APBC

The persistence length for our (infinitely) long sequences of dinucleotides can be determined by various experimental and theoretical studies.^28^ In Figure 4, we present the results of all-atom MD simulations with APBC of *N* = 10 bp segments using the six cases of repeated dinucleotides. Technical details of these MD simulations are given in Appendix B.

To analyze our MD results, we associate a unit orientation vector **h**_*i*_ with each base pair, i.e., *i* = 1, 2, ..., *N*, where *N* = 10*n* is the total number of simulated base pairs. Denoting the angle between the *i*th and (*i* + *j*)th base pair as ϕ_*j*_, we have cos(ϕ_*j*_) = **h**_*i*_ · **h**_*i*+*j*_, which we calculate for all *i* = 1, 2, ..., *N*. Averaging the calculated results over all possible values of *i*, we have
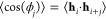
18where the accuracy of this average is further improved by calculating it as a time average over relatively long MD time series. More precisely, we calculate three independent time series of length 10 ns and sample our results every 10 ps, disregarding the beginning of each simulation as the time required to equilibrate the system, see Appendix B for more details. Considering *N* = 10 (i.e., *n* = 1), we plot the averages in eq 18 in Figure 4a for values *j* = 1, 2, 3, 4, 5. We note that
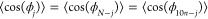
because we use APBC. In particular, the values of the averages in eq 18 for *j* = 6, 7, ... are already represented in Figure 4a by the corresponding values for *j* = 1, 2, 3, 4, 5. In Figure 4, we observe that the results are clearly sequence-dependent for *j* = 1, with the *j* = 4 case providing the best match to the *j* = 1 case. On the other hand, the results are less sequence-dependent for *j* = 2 or *j* = 5. Given the APBC, there is no variation for *j* = *N* = 10 as we have already observed for the WLC model because of the constraint given by eq 12. In Figure 4b, we present the average separation between the subsequent base pairs for each of the studied case.

Our MD simulations in Figure 4 use the smallest possible value of *N* (corresponding to *n* = 1), while one can expect that the results of all-atom MD simulations should be less influenced by the APBC for larger values of *n* (in theory, the APBC-induced errors should decrease to zero in the limit *n* → ∞). To investigate this further, we study the dependence of our results on *n* for the poly(A) case in Figure 5a. We use three independent MD simulations for *n* = 1, 2, 3, 4, 5, 10 corresponding to simulations with *N* ranging from 10 to 100 bp. In each case, we plot the averages given by eq 18 for *j* = 1, 2, ..., 10. We note that this average is trivially equal to 1 in the case *j* = 10 for *N* = 10 bp (because the 1st and the 11th base pairs are identical for *N* = 10 bp), so we omit this artificial value from our plot for 10 bp in Figure 5a. We observe that the results for *n* = 1, 2, 3, 4, 5 are matching some trends of the results for 100 bp. In particular, we can make similar conclusions as in Section 2 that the local properties (smaller values of *j*) are less influenced by using APBC than the averages estimated over the whole simulated polymer length (for *j* comparable to *N*). In Figure 5b, we present the average separation between the base pairs. Comparing with Figure 4b, we observe that the separation between the base pairs is more influenced by the sequence of nucleotides (in Figure 4b) than by using APBC, with only 2–3% variations as we go from 10 bp to 100 bp APBC simulations in Figure 5b.

In Figure 3, we have considered the WLC model with *N* = 100 segments while varying the domain length *L*_*z*_. In Figure 6, we present the results of a similar study using all-atom MD simulations with *N* = 100 bp. The middle bars in Figure 6a,b correspond to the results of the poly(A) case with 100 bp, which has already been included in Figure 5. Using eq 17, this corresponds to *L*_*z*_ = 337.5 Å. The other simulations correspond to the same setup where we either extend or shrink the value of *L*_*z*_ by 5%, i.e., we use the values of *L*_*z*_ given as
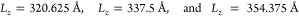
19In Figure 6b, we observe that the average separation between base pairs increases as we increase *L*_*z*_. On the other hand, the behavior of averages in eq 18 is less monotonic as we stretch or shrink the DNA chain, see Figure 6a. Another way to visualize the results of all-atom MD simulations is to consider the average in eq 18 as a function of the distance between the base pairs,^20^ which is visualized as function ⟨*H*⟩ in Figure 6c. To calculate ⟨*H*⟩, we average ⟨**h**_*i*_·**h**_*j*_⟩ over all pairs *i* and *j* such that the corresponding base pairs are the distance *d* apart. We present this average, ⟨*H*⟩, as a function of the distance *d* in Figure 6c. The rate of decay of function ⟨*H*⟩ with distance *d* can be used as an alternative way to define and estimate the persistence length from MD simulations.^20,27^ For example, Kameda et al.^20^ fit the subset of calculated data points ⟨*H*⟩ corresponding to *d* in subinterval 3 ≤ *d* ≤ 100 [Å] by function *f*(*d*) = exp [−*d*/*a*_p_], where *a*_p_ is the parameter defining the persistence length in their study (see Appendix B.3 for more details). Since *L*_*z*_ given by eq 19 are all larger than 100 Å, we can use our data points ⟨*H*⟩ given in Figure 6c to repeat the analysis of Kameda et al.^20^ We obtain that the parameter *a*_p_ is equal to 60.9, 78.0, and 57.6 nm for the 95, 100, and 105% cases, respectively.

The DNA persistence length has been experimentally determined in the force extension measurements^23,29^ to be around 50 nm at the physiological ionic concentrations. This has also been confirmed by different computational studies.^30,31^ The dependence of the DNA elastic properties on the sequence of nucleotides has been studied both computationally^30^ and experimentally.^28^ The experimentally determined persistence length for our sequences of dinucleotides used in Figure 4 have been obtained in the literature^28^ as 50.4 nm (poly(A)), 41.7 nm (poly(C)), 42.7 nm (poly(AT)), 49.6 nm (poly(CG)), 50.7 nm (poly(AC)) and 52.6 nm (poly(AG)). In Figure 4, we have used relatively short DNA segments (10 bp) and calculated ⟨cos(ϕ_*j*_)⟩ defined in eq 18. This value cannot be directly used, for *j* = 1, in eq 16 to estimate the DNA persistence length because nucleic acids are mechanically softer at the scale of a few base pairs^32^ and the WLC model is not applicable at this scale. On the other hand, if we use the value of ⟨cos(ϕ_10_)⟩, which is the average cosine of the angle between the base pairs separated by one helical pitch in formula 16, together with the length of the helical pitch as the length δ of the segment, then formula 16 gives the values of the persistence length around 50 nm as reported in the experimental studies.^28,29^ To get a better fit with the experimental data, the WLC model can be extended to the nonlocal twistable WLC model.^32^ The standard WLC model is the best applicable to larger segments of micron-sized molecules.^24^

### 3.2 Ion Atmosphere

The APBC are useful for investigating solvent properties in the direction perpendicular to the polymer chain. In Figure 7, we present the results of such a study, calculating the radial distribution of K^+^ and Cl^–^ ions. We use three different concentrations of KCl, namely, 0.25, 0.5, and 1 M. In each case, we use *n* = 1, i.e., we use the APBC with 10 bp of poly(A) dsDNA. The results are calculated by averaging over four independent MD time series, each calculated for 10 ns. After the initial transient (of 1 ns) and at equidistant time intervals of 10 ps, we calculate the distance of each ion from the nearest atom of DNA, so our raw data are given in terms of the histograms
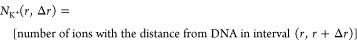

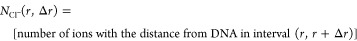
To get the radial distribution function, these numbers have to be divided by the volume, *V*(*r*, Δ*r*), giving the volume of all points that have their distance from the DNA in the interval (*r*, *r* + Δ*r*). Then, the radial distribution of K^+^ ions and Cl^–^ ions is theoretically defined by
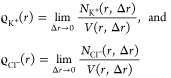
20where *r* is the distance from the DNA. To calculate Figure 7, we approximate the limit in eq 20 by choosing (relatively small) value Δ*r* = 1 Å and we approximate the DNA as a straight line (or equivalently as a straight cylinder) in the *z*-direction, that is, *V*(*r*, Δ*r*) = 2π *r* Δ*rL*_*z*_, giving
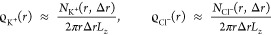
21Formulas 21 are visualized in Figure 7 as histograms. We observe that the concentration of K^+^ ions is higher in the vicinity of DNA because positive ions preferentially visit electronegative sites around DNA, although direct binding of ions to DNA bases is a relatively rare event, as previously reported in the literature.^4^

## 4 Discussion and Conclusions

Using MD simulations at constant pressure and temperature, we can solvate the DNA with water and ions, fixing the concentration of ions in the bulk. In Section 3.2, we have presented illustrative results of such all-atom MD investigations with APBC. Such simulations can also be used to estimate other solvent properties, for example, the moments of force distributions on ions, which can be used for parametrizing coarse-grained stochastic models of ions used in multiscale and multiresolution simulations.^16,19^ The APBC simulations can also be coupled with coarse-grained models of water to design adaptive resolution simulation techniques.^5,33,34^ In Section 3.2, we have presented the results calculated with APBC using *n* = 1 helical pitch. In particular, the simulated domain length is around 3.4 nm long and considerably smaller than the DNA’s persistence length, which is about 50 nm. To study the mechanical properties of DNA, we need to increase the number of helical pitches as we have shown in Section 3.1 with our MD simulation results considering up to *n* = 10 helical pitches along the *z*-direction of the APBC simulation domain given by eq 1.

To get further insight into the correct use of the APBC, we have started our investigation using a discrete worm-like chain (WLC) model in Section 2, where we have observed in Figure 3 that the APBC affect less some local properties of the polymer chains than some global averages. In particular, the persistence length of the polymer chain can be estimated from the local properties of relatively short polymer chains, simulated with the help of APBC. The APBC are also applicable to simulations of biopolymers with larger persistence lengths (for example, actin filaments^35,36^) when a modeler is interested to understand the properties of the surrounding solvent. In Section 2, we have introduced the WLC model in the discretized form, where the polymer chain energy, given by eq 2, only includes the bending energy. Equation 2 is applicable to modeling a WLC polymer in the three-dimensional physical space  without any applied force. Such a model is analyzed in Section 2.1. In Section 2.2, we consider the WLC model with the APBC, which can be either viewed as introducing the additional holonomic constraint given by eq 12 or as stretching the polymer chain using the corresponding constraint force. In particular, the latter interpretation of the APBC also means that the polymer chain energy includes not only the bending energy given by eq 2 but also the corresponding stretching energy.^23,24^ This viewpoint can be used for the analysis of the WLC model with constraints, although many analytical studies use a simplified eq 12, which is restricted to the *z*-direction, where the studied problem is effectively one-dimensional.^37^ In the case of the APBC, we do have the constraints in the *x*-direction and *y*-direction as well, given by eq 12; see Appendices A.2 and A.3 for more details on the analysis and simulations, respectively, in this case.

In Appendix B, we provide the technical details of all-atom MD simulations, including the treatment of constant pressure simulations. The barostat used is again asymmetric with no fluctuations of *L*_*z*_. In the APBC simulations, we have different treatments of the *z*-direction and all perpendicular directions in the *x*–*y* plane. Simulations with 2D periodicity have also been used to study the behavior of a slab of water between two metallic walls,^38^ which can be treated using three-dimensional Ewald techniques by including the image charges. One advantage of the APBC simulations is that they can be implemented with relatively minor modifications of standard all-atom MD tools,^39−43^ as detailed in Appendix B. We note that the number of simulated base pairs, *N*, and the number of helical turns, *n*, in the DNA model with APBC has been fixed to satisfy *N* = 10*n* in all presented MD simulations, and thus the model does not allow for over-winding or under-winding of DNA.^44^ This can be studied by relaxing the assumption *N* = 10*n* in all-atom MD simulations, and by extending our basic WLC model to twistable WLC models.^32,45^

## A. WLC Model: Additional Derivations and Simulations

Here, we provide additional details needed for the derivation of eqs 8, 9, and 15, and further details behind the Monte Carlo simulations in Figure 3.

### A.1 Derivation of Equations 8 and 9

Considering small values of α ≪ 1, we have . Substituting into eq 6 and evaluating the resulting integrals, we obtain

Substituting this expansion into eq 16, we obtain eq 9 in the limit α → 0. On the other hand, if α ≫ 1, we can use the double angle formula cos(θ)sin(θ) = sin(2θ)/2 and substitution *y* = θ√α to deduce

where replacing the upper integration limit by ∞ only results in an exponentially small (negligible) error. Next, considering the limit α → ∞, we apply the Taylor expansion of the sine function, sin(*x*) ≈ *x* – *x*^3^/3! + *x*^5^/5! – *x*^7^/7!, and evaluate the resulting integrals to obtain

22

Similarly, using the substitution *y* = θ√α, we get

23

Substituting eqs 22 and 23 into 6 and dividing the power series, we obtain

as α → ∞. Substituting this expansion into eq 16, we obtain eq 8 in the limit α → ∞.

### A.2 Derivation of Equation 15

Since  for *N* = 2 and *N* = 3, eq 15 is satisfied for *N* = 2 and *N* = 3. Considering a general value of *N*, the constraint in eq 12 can be rewritten as

Taking the scalar product of this equation with **l**_1_, we get

24

where we used ⟨**l**_1_⟩ = [0, 0, *L*_*z*_/*N*]. Assuming that ⟨**l**_1_·**l**_*i*_⟩ are equal to each other on the left-hand side of eq 24, we get  for *i* = 2, 3, ..., *N*. Substituting into eq 3, we obtain

which is eq 15. Considering large values of *N*, eq 15 simplifies to

which can also be deduced from eq 24 by considering that the left-hand side of eq 24 is twice the sum needed for the calculation of .

### A.3 Monte Carlo Simulations of the WLC Model

In Figure 3, we present some illustrative results estimated using Monte Carlo simulations of the WLC model with *N* = 100 segments for different values of the domain length, *L*_*z*_, and the stiffness parameter, α. We use *L*_*z*_ ∈ {10, 20, ..., 90} and α ∈ {0, 1, 5, 10}. Given *L*_*z*_ and α, our results are sampled over a long Monte Carlo trajectory consisting of 10^10^ steps. To implement APBC, we start with an initial configuration of the polymer satisfying eq 12. In our implementation, the initial *z*-coordinate of the *i*th polymer segment is equal to *iL*_*z*_/*N*, for *i* = 1, 2, ..., *N*, while its *x*- and *y*-coordinates are chosen to be equal to  . Then, the length of each segment is equal to δ and the eq 12 is satisfied. However, our results are independent of our specific choice of the initial configuration because we use the first 10^6^ steps to equilibrate our polymer configuration, i.e., we evolve the system over 10^6^ steps without saving any data. Then, we use 10^10^ steps to sample our results. In each Monte Carlo step, we select (a possible) new configuration of the polymer by choosing two nonadjacent beads at random (say the *i*th and *j*th bead) and we rotate the part of the polymer between the *i*th and *j*th bead along the line connecting these beads. We rotate it by a random angle. In this way, we make sure that the new configuration of the polymer satisfies the eq 12. The updated configuration is then either accepted or rejected using the standard Metropolis–Hastings criterion^26^ based on the chain bending energy given by eq 2.

## B. All-Atom MD Simulation Details

Throughout the all-atom modeling and simulation, we used CHARMM36 force field, VMD 1.9.3 (for modeling),^39^ and NAMD 2.14 (for simulation).^40^

### B.1 APBC Implementation

The periodic DNA models (*N* bp) used for the APBC are generated as follows. First, an (*N* + 1) bp long dsDNA configuration (in PDB format) is constructed with 3DNA (Web 3DNA 2.0^41,42^) in such a way that the (*N* + 1)th base pair is equivalent to the first base pair translated to the *z*-direction. Here, we use the base pair step parameter set for B-DNA (calf thymus; generic sequence) with Twist 36.0 degrees and Rise 3.375 Å for all nucleotide sequences. Note that the resulting structure shows a slight (sub-ångström) shift between the first and the (*N* + 1)th (i.e., deleted) bases depending on the length and nucleotide sequence, which is then relaxed in the initial equilibration simulation run. Then, a nucleotide at the 3’-end of each strand is removed, and the corresponding structure (in CHARMM PSF format) is generated by AutoPSF. Finally, by editing the PSF file, the bond to the 3’-end (removed) nucleotide is substituted with that to the first nucleotide (i.e., 5’-end), and the angles and dihedrals (4 and 7 terms for each strand, respectively) are reconnected accordingly. These additional terms are listed in Table 1. Note that, for the CHARMM36 force field, the type of the 5’-end phosphate (usually the first atom of each strand) should be changed from P to P2, as it is no longer at the terminal with the APBC.

**Table 1:** List of Additional Terms Connecting the Ends of Each DNA Strand

bonds			
*N*th O3’	1st P		
angles			
*N*th C3’	*N*th O3’	1st P	
1st O1P	1st P	*N*th O3’	
1st O2P	1st P	*N*th O3’	
1st O5’	1st P	*N*th O3’	
dihedrals			
*N*th C4’	*N*th C3’	*N*th O3’	1st P
*N*th C2’	*N*th C3’	*N*th O3’	1st P
*N*th C3’	*N*th O3’	1st P	1st O1P
*N*th C3’	*N*th O3’	1st P	1st O2P
*N*th C3’	*N*th O3’	1st P	1st O5’
*N*th H3’	*N*th C3’	*N*th O3’	1st P
*N*th O3’	1st P	1st O5’	1st C5’

For simulation with explicit solvent, each model has been solvated in an *L*_*x*_ × *L*_*y*_ × *L*_*z*_ TIP3P water box, where *L*_*x*_ = *L*_*y*_ = 200 Å and *L*_*z*_ = 3.375*N* Å, then neutralized by K^+^ and 0.15-1M KCl has been added in the bulk.

### B.2 Molecular Dynamics Simulation

After 10^4^ steps of energy minimization, each model was simulated for 10 ns at 2 fs time step (with SETTLE method for hydrogen). Nonbonded interactions were cut off at 12 Å (with switching from 10 Å), and particle mesh Ewald method was used for electrostatics. Langevin thermostat at 300 K (damping: 1 per ps) and Nosé–Hoover Langevin piston barostat at 1 atm (period: 2 ps, decay: 1 ps) were applied. For the barostat, constant area setting was used, keeping *L*_*z*_ (and *L*_*x*_) constant and adjusting only *L*_*y*_ to control the pressure. The atomic positions were recorded at 10 ps intervals and used for analysis.

For simulations with periodic box length with different values of *L*_*z*_ in Figure 6, the *z*-coordinate of each atom in the DNA model was rescaled and then solvated into the water box with the adjusted value of *L*_*z*_.

### B.3 Analysis

The DNA structure in each snapshot was processed by 3DNA.^42^ To consider also the base-pair step crossing the periodic boundary (i.e., the *N*th to the first base pair), a periodic image of the first residue (shifted by *L*_*z*_) was added at the end of each strand, and the resulting (*N* + 1) bp long segment was processed by 3DNA. The subsequent analysis then used the helical-axis positions and helical-axis vectors providing **h**_*i*_ in eq 18. The distance between adjacent base-pair steps was obtained as the distance between the corresponding helical-axis positions, and other distances along the chain are calculated as the sum of those adjacent distances within the section. These values were averaged over three simulation trials except the initial 1 ns (Figures 4, 5, and 7) or 5 ns (Figure 6) for each case.

To calculate ⟨*H*⟩ in Figure 6c, we average ⟨**h**_*i*_·**h**_*j*_⟩ over all pairs *i* and *j* such that the corresponding base pairs are distance *d* apart. More precisely, the distance *d* is divided into intervals [*d* – Δ*d*/2, *d* + Δ*d*/2) with width Δ*d* = 0.1 Å, and *d* takes values of multiples of Δ*d*. To estimate the persistence length *a*_p_, the data points ⟨*H*⟩ in the subinterval 3 ≤ *d* ≤ 100 [Å] (the same setting as in Kameda et al.^20^), accumulated over the three trials for each case, are fit by function *f*(*d*) = exp[−*d*/*a*_p_] using the method of least squares weighted by the number of samples in each bin.
